# Endoscopic endonasal resection of giant pediatric craniopharyngioma: impact on hypothalamic edema

**DOI:** 10.3171/2020.4.FocusVid.19926

**Published:** 2020-04-01

**Authors:** Mina M. Gerges, Brett Youngerman, Vijay K. Anand, Jeffrey P. Greenfield, Theodore H. Schwartz

**Affiliations:** Departments of 1 Neurosurgery,; 2 Otolaryngology, and; 3 Neuroscience, Weill Cornell Medicine, NewYork-Presbyterian Hospital, New York, New York

**Keywords:** endoscopic, endonasal, transsphenoidal, minimally invasive, craniopharyngioma, pediatric, hypothalamus, surgical video

## Abstract

An 8-year-old child presented with fatigue, weight loss, and visual deterioration. MRI demonstrated a craniopharyngioma with compression of the optic chiasm and extensive edema on the hypothalamus and optic radiations. The tumor was completely removed via an endoscopic endonasal approach. Postoperatively, vision improved and hypothalamic edema completely resolved within 5 days. This video demonstrates the technical nuances of the surgery and discusses the impact of surgery on the hypothalamic nuclei in pediatric patients.

The video can be found here: https://youtu.be/wxkBmhTPi6c.

## Transcript

This video will document the endoscopic endonasal resection of giant pediatric craniopharyngioma and also discuss the issue of hypothalamic invasion and impact of surgery on hypothalamic edema.

**0:33** This 8-year-old girl presented with 2-month history of fatigue, early satiety with 5 pounds weight loss, blurry vision, and falling off of her growth curve.

**0:42** CT and MRI revealed very large solid and cystic craniopharyngioma with hydrocephalus, and a calcified and cystic component consistent with adamantinomatous craniopharyngioma. There was extensive edema within the hypothalamus and the optic radiations, which raises the question of whether the tumor should be completely removed for cure or just debulked to avoid damaging the hypothalamus but necessitating postoperative radiation therapy.

**1:05** In order to relieve her hydrocephalus, intraventricular endoscopic biopsy and cyst fenestration were performed. Visual improvement was noted as well as improvement in hydrocephalus. Patient went home to trick-or-treat for Halloween and then returned 10 days later for definitive resection of the tumor.

**1:15** The questions to be addressed are, which approach to choose, endonasal or transcranial; how does edema in hypothalamus influence the decision; and what are the key steps of surgery and the closure?

In extensive review of literature comparing endonasal to transcranial surgery for craniopharyngioma, it is demonstrated that gross-total resection rates are similar, CSF leak rates now comparable as well; however, visual improvement is markedly higher following the endoscopic endonasal approach.^4^ For this reason, we chose to perform an endoscopic endonasal approach for this child. Our results in adults have shown gross-total and near-total resection in approximately 95% of patients and CSF leak rate about 3%. However, in pediatric cases, resection of tumor in hypothalamus has been shown to increase in BMI. In these studies, the rate of recurrence after subtotal resection and radiation are not adequately documented as there is much shorter follow-up in subtotally resected patients and the authors used reoperation as a surrogate for recurrence, so cases lost to follow-up or who go to other institutions or pass away are not included.^2,3^ In addition, when examining the hypothalamic nuclei involved in satiety and body weight, they are found in the lateral walls of the ventricle, not the floor. In our prior study of endonasal endoscopic resection of pediatric craniopharyngiomas, BMI increased in only 20% of the patients despite a 50% rate of gross-total resection.^1^

**2:50** Craniopharyngiomas can be found in sella, sellasuprasellar or only suprasellar, as well as only in the third ventricle. The key surgical steps are first to sweep the superior hypophyseal arteries laterally and superiorly to preserve the blood flow to the chiasm and dissect under the chiasm to expose the plane between the tumor and hypothalamus and preserve the lateral wall of the third ventricle, remove the superior extent of the tumor, preserve the stalk as long as possible, and then sacrifice the stalk if required to achieve a gross-total resection.

**3:16** First, the endonasal endoscopic approach is performed. The ostia are identified bilaterally. Bilateral nasoseptal flaps are elevated, the posterior septum is removed, a bilateral sphenoidotomy is performed. Given this is a pediatric patient, extensive drilling is required to enlarge the sphenoid sinus to create large enough corridor for surgery. This involves opening up the anterior wall, resecting superior turbinate bilaterally, and performing posterior ethmoidectomies. We try to save the middle turbinates as much as possible and only resect the turbinate on the left if we need more room for the scope.

Next, we open up the sella and the tuberculum with diamond drill and Kerrison in order to make an adequate opening. Doppler is used to identify the blood vessels. An opening is made in the bond from the top of the sella to just above the chiasm in the tuberculum. The dura is opened. A large sellar craniopharyngioma is identified and this first internally decompressed, and then we start to dissect the tumor from the surrounding neurovascular structures, first preserving the superior hypophyseal arteries. Sweeping movement was used to dissect the arachnoid off the lateral region of the tumor away from the PComm. Inferiorly, the basilar artery can be identified in the inferior aspect of the tumor. We also can identify the mammillary bodies which have to be preserved. Here we are sharply dissecting the posterior aspect of the tumor off the mammillary bodies. Here we are mobilizing the tumor away from the hypothalamus and then additional internal decompression is done. The plane between the tumor and the hypothalamus/lateral walls of third ventricle is very carefully dissected under direct vision. Normal hypothalamus is swept laterally; this also done with the mammillary bodies inferiorly sharply using microscissors if the plane is unclear. Again, the lateral walls more superiorly are dissected free from the tumor under direct vision to expose the lateral walls of the third ventricle/medial hypothalamus and superior hypothalamus in both sides. The tumor extends superiorly and the superior wall is equally and carefully and bimanually dissected free from the hypothalamus in order to minimize any damage to the hypothalamus, which is the goal of surgery in this pediatric patient. The tumor is then removed underneath the chiasm. Inspection shows that the lateral walls are intact with some petechial hemorrhage.

**6:07** The closure was performed with AlloDerm and gasket seal using rigid buttress to keep it in place and vascularized nasoseptal flaps for final coverage, held in place with tissue seal.

**6:21** Postoperative MRI scan reveals gross-total resection of the tumor, but most interestingly, in this case, that the edema in the hypothalamus and optic tracts in the MRI scan 5 days postoperatively was gone, raising the question of whether surgery to remove the tumor completely is not a better strategy to preserve hypothalamic function than subtotal resection leaving tumor invading the hypothalamus followed by radiation.

**6:50** The patient recovered well and her vision improved. The lumbar drain was removed in 2 days. She was cognitively intact with panhypopituitarism and went home on postoperative day 6.
